# Target capture strategy selection in a simulated marksmanship task

**DOI:** 10.1038/s41598-019-50551-z

**Published:** 2019-10-01

**Authors:** Noah J. Steinberg, Alexander A. Brown, Luis F. Schettino

**Affiliations:** 1 Lafayette College, Department of Psychology, Easton, PA USA; 2 Lafayette College, Department of Mechanical Engineering, Easton, PA USA

**Keywords:** Neuroscience, Psychology

## Abstract

This paper examines how individuals track targets that move in relatively unpredictable trajectories. Gaze and behavioural data were captured as twenty two participants learned a simulated competitive marksmanship task known colloquially as the Death Star over six training days. Participants spontaneously selected one of two consistent target-tracking strategies with approximately equal probability. Participants employed either chasing behaviour, in which gaze follows a target’s trajectory before a shot, or ambushing behaviour, wherein gaze anticipates the trajectory and the participant intercepts a moving target predictively. All participants improved in task performance measures (completion time and number of shots), but did so at the expense of accuracy in missed shot attempts. Surprisingly, neither behavioural strategy offered a significant advantage in task performance measures, indicating that either may be equally effective in tackling a hand-eye coordination task with complex target motion such as the Death Star.

## Introduction

To interact effectively and efficiently with moving objects in the environment, humans must be able to track them visually with a high degree of accuracy, interpret their motion, and coordinate limb movements efficiently. Catching a ball is a classic example, but some tasks involving moving objects are far more complex, such as swerving to avoid a deer in the road.

As objects shift their locations in space, gaze is displaced to maintain the object within the fovea, the region of the visual field with the highest level of acuity^1,2^. Critically, successful tracking and interaction with moving objects depends fundamentally on the ability of individuals to predict future target locations within relatively small temporal windows^3^.

Early work demonstrated that the ability to visually track simple target motion can be learned readily^4,5^. Leung and Kettner^6^ showed that non-human primates are able to track targets in complex 2D paths. This indicated that it is possible to produce highly accurate predictive eye movements beyond the tracking of simple trajectories.

While the effects of learning on gaze control indicate that individuals can select and track targets moving in complex trajectories, how they do so is less clear. Specifically, it is not clear whether all individuals, when asked to interact with moving targets in a hand-eye coordination task, will converge on a similar strategy. For example, once they have selected a target, subjects could track its path through space prior to interacting with it in a chasing type of behaviour^7^. Conversely, based on experience, participants could also select a region of space where targets can be expected to appear, prior to executing a movement. This ambushing approach to target capture is a strategy akin to interceptive actions^7,8^.

Evidence from sports such as Cricket^9^ and Table Tennis^10^ supports the idea that in tasks involving an incoming target, individuals predict its future location and their eyes saccade to it. This is a strategy suggestive of ambushing behaviour. Nonetheless, research on target interception in a number of sports has suggested that participants may adopt chasing^11^ or, more commonly, a mix of the two types of strategies^7,9,10,12,13^. The characteristics of some of these tasks such as target size, trajectory and speed may impose strong constraints on the type of strategy chosen. However, it is not known whether participants select one over the other spontaneously in tasks where target motion is more complex than that experienced in the standard eye-hand coordination activities found in the literature.

In laboratory tasks that involve relatively unpredictable motion in hand-eye coordination tasks, such as in the work of Mrotek and Soechting^14^, participants have been known to either intercept a target by either moving the hand in front of the target or “catching up” to the target, which also indicates that a dichotomy in strategy may exist. Mrotek and Soechting^14^ required participants to capture a target by moving a finger over it on a touch screen as it moved in a cyclical but complex trajectory. Based on an analysis of movement initiation times and target motion during interceptive movements, they concluded that subjects predicted roughly 150 milliseconds of target motion, and that the initiation of an interceptive movement was tied to certain characteristics of target motion. While the authors noted anecdotally that some participants varied in the way they approached targets in ways suggestive of either chasing or ambushing behaviour, they did not pursue this trend as part of their study. In a similar experimental setup, Danion and Flanagan^15^ found that when subjects needed to track targets moving in complex trajectories with both hands and eyes, smooth pursuit with the eyes became more dominant than saccadic movements, as evidenced by a decrease in “catch-up saccades” when target motion was tracked with both hands and eyes, rather than with eyes alone. This seems to suggest that “chasing” behaviour, in which subjects follow targets rather than “skip ahead” of them and wait for them to arrive, might be more common in hand-eye coordination tasks with complex target trajectories. Still, the question of to what extent the dichotomy observed in Mrotek and Soechting^14^ would appear in a more ecological target capture task with increased performance demands is yet unsolved.

**Figure 1:**
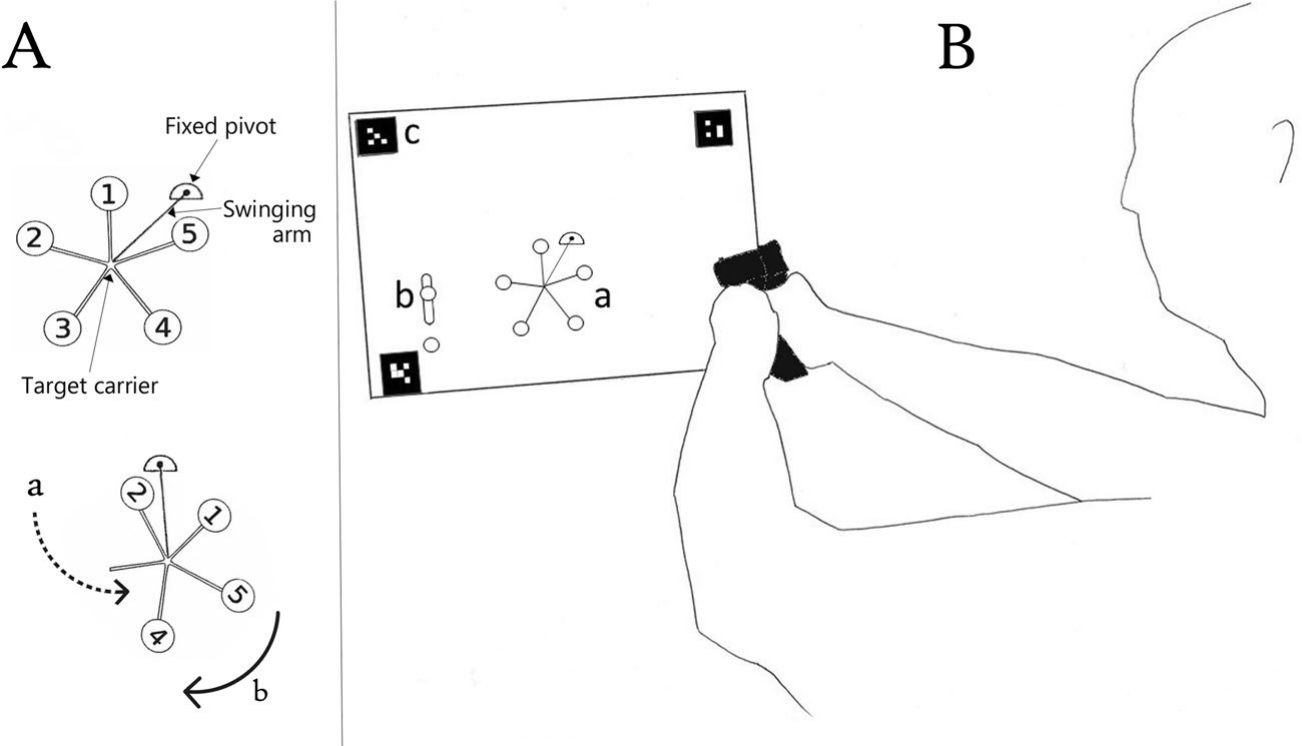
(**A**) Elements of the Death Star. Once a trial starts, the star begins a pendular movement on its swinging arm (a, dashed arrow). When one of the targets is hit, the balance of the star changes, initiating a rotational movement around the target carrier (b, solid arrow). (**B**) Experimental setup. Participants shot a projection of the DS (a) using a plastic pistol fitted with a laser pointer from a distance of 3 meters. Simulation began after a successful shot to the activator target (b). Surface tracking markers (c) allowed for proper alignment between the projection and the eye tracker.

Due to their strong eye-hand coordination requirements, marksmanship tasks offer an ideal means to test gaze-control strategies and hand-eye coordination^16,17^. Tasks involving moving targets, such as clay target shooting with a shotgun, require participants to track and select (capture) moving targets in the form of flying clay discs^11^. However, in traditional clay target shooting, targets move along relatively simple curvilinear trajectories, making target motion similar to that in constant velocity laboratory tasks (such as those employed by Tresilian^7^). Brown^18^ recently developed a simulated version of the marksmanship task known as the Death Star (DS). The DS, used in professional three-gun competition, consists of five steel plates located at the ends of a five-pointed, star-shaped carrier. The carrier rotates on a bearing at its centre, and is attached to an arm that swings about a second, fixed pivot point (see Fig. 1). With all five steel plates attached to the carrier, the star swings about the fixed hinge like a simple pendulum. However, when even one plate is shot from the carrier, the star’s inertia changes, resulting in complex behaviour similar to that of a so-called “double pendulum”. Specifically, the DS’s sensitivity to initial conditions means that no two trials are exactly alike, and that the selection of targets in a particular order can have a substantial effect on target trajectories. In spite of this, competitors learn to master this task efficiently, suggesting that, at least momentarily, target motion is predictable. Moreover, certain shooting strategies can modulate the Death Star’s capacity for unpredictable motion, favouring improved performance. Based on anecdotal evidence, Brown^18^ suggested that in order to perform successfully on marksmanship tasks such as the DS, individuals may employ a mix of chasing and ambushing strategies.

The present experiment was designed to determine whether nave participants, during the learning of the DS task, would choose a chasing, ambushing or a mix of gaze control strategies and whether the type of strategy selected would affect success rate. Participants practised on the DS simulation over six training days. To test the gaze control strategy used, we compared the velocity vectors of the eye relative to those of the target of interest within a 150 ms window prior to a successful shot, as this time window is crucial for successful target interception^13,19^ and is commensurate with the prediction horizon suggested in Mrotek and Soechting^14^.

We predicted that, as training progressed, participants would prefer a chasing strategy more often than an ambushing strategy, with eye and hand following the targets closely as in Danion and Flanagan^15^. We also predicted that following the targets closely would permit more robust target capture than an ambushing strategy, given the complex nature and unpredictability of the task.

## Methods

### Participants

Twenty-two participants (6 males, ages 18–22) free of neurological disorders took part of the study. Participants were recruited from the subject pool at an undergraduate institution. All participants had normal or corrected-to-normal vision. Participants with corrected vision were required to wear contacts to prevent their glasses from interfering with the eye-tracking headset used to record gaze information. In a brief survey, participants verbally reported they had no experience with marksmanship prior to the experiment and provided information regarding their previous experience in eye-hand coordination activities, including racket and team sports and the level of competition.

All participants read and signed informed consent forms approved by the IRB of Lafayette College. The experiments were conducted in accordance with the ethical standards laid down in the Declaration of Helsinki. Experimental protocols and procedures were approved by the IRB of Lafayette College.

### Materials

Participants shot a simulated DS target using a plastic pistol outfitted with a laser pointer (NLT SIRT 107, Next Level Training, Cedar Knolls, NJ). The pistol contained a lead weight to mimic the weight of an actual pistol used in competition. The pistol’s red laser was visible to the participants when they shot. Gaze and DS target time-series information were saved as text files after every successful trial and were analysed using custom written Matlab (The Mathworks, Natick, MA) programs. A successful trial was defined as a trial in which all five targets were hit in any order. Because no limits were imposed on Trial Length, there were very few non-successful trials. The only circumstance that qualified as a non-successful trial was if an individual shot a target before shooting the static, oblong activator target (see Fig. 1), which initiated the movement of the DS, as well as the internal timer. Bad trials were discarded.

#### Task simulation

We employed an open source target simulator written in Python by Brown^18^. The simulation was projected via a Gigabyte ‘mini-box’ style PC with a built-in projector running Ubuntu Linux. Laser hits were measured using a Microsoft webcam modified with a near infrared-pass filter that was permanently affixed to the PC. The simulation thread of the software computed the dynamics of the DS target at 60 *Hz* and the target was projected with a resolution of 480 × 720 pixels. The projected simulation area was 90 *cm* high by 130 *cm* wide, resulting in a version of the DS target at approximately $\frac{1}{3}$ scale. Specifically, each projected target plate had a diameter of 4.5 *cm*, the projected target carriage radius was 31.5 *cm*, and the projected swinging arm’s length was 28.3 *cm*. Because participants were performing the task from 3 *m* in front of the projection surface, and the target was projected at $\frac{1}{3}$ scale, the task simulated engaging a real DS target from 9 *m*.

A second thread in the simulation software processed images from the built-in webcam at roughly 30 *Hz*, detecting laser hits using a brightness threshold-based algorithm. To compensate for the image acquisition delay in the simulation’s hit detector, the centroid of each laser hit was compared to a time-delayed record of each target’s position, using a measured mean image processing delay value of 150 *ms*. To mitigate the risk of false negatives due to variations in hit detection due to lighting variations and/or camera calibration issues, the simulation employed a small built-in spatial allowance to determine a ‘hit’ of 2 times the radius of the projected target’s delayed position. This allowance was chosen heuristically by manually checking hit detection accuracy on a stationary target across the full range of possible positions on the screen. The system recorded date-and-time stamped position, velocity, and shot data. Output text files also contained information about whether a shot was detected and its pixel location at each simulation timestep.

#### Eye tracking

A binocular eye-tracking device (Pupil Labs, Berlin, Germany) was employed to record pupil and gaze information. The headset’s outward facing camera had a resolution of 1280 × 720 pixels and a refresh rate of 60 *Hz*. Each of the eye-facing cameras had a resolution of 640 × 480 pixels, and a refresh rate of 120 *Hz*. Data capture, export and pre-processing was conducted with the open source Pupil Labs software (Pupil Labs, Berlin, Germany). Pupil Player’s surface detection feature was used to obtain gaze information in allocentric coordinates by tracking four Aruco Markers^20,21^ forming a 110 *cm* high by 150 *cm* wide rectangle on the projection surface, with dimensions chosen to fully encompass the task simulation’s projection. The Pupil system provides accurate gaze estimates (0.6°, 0.08° precision) and a processing latency of 0.045 *s*^22^.

#### Spatiotemporal data alignment

Gaze data and DS simulation data were spatially and temporally aligned prior to further analysis. The two data sets were time-synchronised for each trial by identifying the video frame in which the activator target (which started the simulation time) was successfully hit. Briefly, gaze data were interpolated to match the temporal frequency of the DS data. A cross-correlation template-matching algorithm was used to detect the video frame of the activator target’s disappearance to identify the frame number to temporally match the simulation and eye tracking data. Finally, allocentric gaze data were smoothed using a zero-phase digital 3^rd^ order Butterworth filter with a cutoff frequency of 6 *Hz*, and all units were converted to visual degrees.

### Design and procedure

All participants were shown how to operate the plastic pistol prior to beginning the task on day 1. Each day participants were reminded of their objective: high performance in the form of high shot accuracy and low trial completion time. Participants were asked to keep their head still as much as possible and to track the targets with their eyes. A successful shot on the stationary activator target (see Fig. 1B) initiated the simulation. Each training session consisted of 3 ‘warm up’ static (the DS did not move) and 20 dynamic trials, which the participants completed at a pace comfortable to them. There were no constraints on the number of shots that could be fired per trial.

Participants came to the laboratory for 30 minutes on five consecutive days (Training Days 1–5) followed by a rest period of two days and a retention test on day 8 (Training Day 6). Prior to beginning the trials, participants were outfitted with the eye-tracking system. Calibration was performed with a 9-point grid calibration and was considered acceptable when participants accurately fixated on all four corners as well as the centre of the simulation screen.

Half of the participants received ‘quiet eye training’ in the form of verbal suggestions and video examples of expert performance in order to determine whether such training would result in differential performance^23^. Statistical analysis showed no effects of quiet eye training on performance, indicating that both groups performed similarly. Therefore, all participant data were pooled for further analysis.

#### Task performance

Trial Length was calculated by subtracting the final timestamp (time when the fifth target was hit) from the first timestamp (time when the activator target was successfully shot). Number of Shots was the shot count in completed trials. Shot count did not include the shot on the activator target. Shot Accuracy was the average Euclidean distance between each shot taken and the closest target. Given that the trials could be shot in any order, there were 120 (5!) possible permutations. We looked at the distribution of shot order per trial (in successful trials) and identified those permutations selected by participants at a rate significantly higher than chance.

#### Gaze control

Saccade analysis was conducted in Pupil Player software using the software’s ‘3D Fixation Detector’, which detects fixations using the pupil’s normal angle for dispersion calculations^22^. The goal of our study was to track participants’ eye motion in relation to target motion in order to characterise their gaze control strategies. However, due to the requirements of our task and the hypothesis we wished to test, previous methods for assessing gaze control were found to be poorly suited. For example, traditional Pursuit Gain (the ratio of eye velocity to target velocity)^24,25^ does not account for situations when an individual saccades towards a temporarily stationary target (which would produce a pursuit gain of infinity). Even when using an extended version of pursuit gain, such as the implementations in Mrotek and Soechting^14^ or in Danion and Flanagan^15^, where the use of the vector inner product between eye and target velocity allowed for the computation of pursuit gain with complex target paths, we found that the numerical issues with computing pursuit gain by dividing by target velocity when the target was nearly stopped could produce very large pursuit gains. This was true even in cases with low eye velocities. This numerical property of pursuit gain could make it relatively difficult to differentiate between a participant who has allowed a target to approach his/her gaze location as the target stops, and one who ‘catches up’ to the stopped target location just before capture, because the divisor of the pursuit gain equation would be nearly zero in either case. Position and Velocity Mean Squared Errors have also been used to characterise gaze in target tracking tasks^26^, but proved unfeasible in our case due to limitations in positional accuracy where mismatches between our projection and gaze data coordinates produced MSE values with a poor Signal-to-Noise Ratio.

In order to cope with these issues, and to develop measures that capture key behavioural characteristics of “chasing” and “ambushing”, we employed two new measures related to gaze behaviour: Final Saccade time and Path-Coordinate Relative Eye Velocity (PCREV). Given that each trial consisted of five targeting and shooting events, we looked at the time when a participant made a saccade towards a target of interest, with time measured relative to the occurrence of their successful shot. This measure allowed us to determine whether the participants were actively searching for a target. In the case of PCREV, we were interested in quantifying the approach of a participant’s gaze to a moving target. PCREV was designed as a measure of whether participants’ gaze is ‘catching up’ to a target or if it is relatively static, waiting for the target to enter the field of view. This measure has some similarity to the ‘direction cosine’ employed in Mrotek and Soechting^14^, which quantified how well target and eye velocity aligned, but PCREV directly measures the difference in the two velocities in the component aligned with the target’s path. We describe these measures in more detail below.

#### Final saccade (FS)

In order to determine whether participants used their gaze to actively search for targets, we normalised (%) the time between each shot (hit or miss) and a successful shot (a ‘pursuit attempt’). We then identified the time in which the last saccade before the successful shot was made within a pursuit attempt. A minimum value of 1 indicates that the saccade was at the beginning of the pursuit attempt while a value approaching 100 indicates it happened later in the attempt. Thus, FS allows us to determine the relative timing of the last saccade towards a target of interest. FS occurring early during a pursuit attempt suggests that the participant’s gaze jumped quickly towards the path of a chosen target, allowing the participant to track (‘chase’) the target leading up to target capture. In contrast, FS occurring late during a pursuit attempt (very shortly before shooting) indicates an attempt by participants to place their gaze at a location where a target is predicted to appear.

#### Path coordinate relative eye velocity (PCREV)

PCREV was chosen as a measure to show the degree to which a participant’s gaze ‘catches up’ to a target in the interval preceding each successful shot by looking at the component of the gaze velocity tangential to the target’s motion at each simulation timestep. This is similar to the direction-sensitive aspects of the pursuit gain and direction cosine measures implemented in^14^, but PCREV is defined fundamentally as the difference between gaze and target velocity in the direction of target motion, rather than the quotient (in the case of pursuit gain).

If a participant’s gaze chases a target, mean PCREV values in the pre-shot interval (150–50 *ms* prior to a shot) should be positive, indicating that a subject is ‘catching up’ to the target just before the shot. Negative PCREV values are likely to indicate ambushing behaviour (where the participant holds his/her gaze at a particular location of the visual field) because the target’s velocity in the direction of target motion is greater than the eye’s, indicating that the target is ‘moving towards’ a participant’s predictive estimate of the target’s position. When the eye and target are moving together, including the case in which the target is stopped or its velocity is very low, PCREV is 0.

To compute PCREV, the filtered gaze positions and raw DS simulation positions for each target were numerically differentiated to obtain velocities in the simulation surface’s XY coordinate system. Then, expressing a pursued target’s velocity vector as ${\overrightarrow{v}}_{t}$ and the gaze velocity vector (projected onto the simulation surface to maintain consistency in units) as ${\overrightarrow{v}}_{e}$, the difference between the component of the gaze velocity in the direction of the target and the target’s velocity, which we call PCREV, is given by Eq. 1.

1 $$PCREV={\overrightarrow{v}}_{e}\cdot \frac{{\overrightarrow{v}}_{t}}{||{\overrightarrow{v}}_{t}||}-||{\overrightarrow{v}}_{t}||$$

This metric assures that for small values of the target velocity’s magnitude $||{\overrightarrow{v}}_{t}||$, the computation for PCREV approaches zero rather than infinity. PCREV values were computed for all timesteps in the pre-shot period, and then averaged for analysis.

### Statistical analysis

Analysis of data was conducted using custom Matlab programs while statistical tests were handled with SPSS statistical software (IBM, Armonk, NY). Efficacy at the DS task was determined through three measures: Trial Length, Number of Shots and Shot Accuracy. Gaze Behaviour was assessed through FS and PCREV.

In order to assess learning of the DS task, repeated measures (RM) analysis of variance (ANOVA) with Training Day (Training Days 1–6) as within-subjects variable (TDAY) were run for all DS variables (Trial Length, Shot Count, Shot Error). Mixed-design ANOVAs with Behavioural Strategy as the between-subjects variable (Chasers, Ambushers) and training day (Training Days 1–6) as the within-subjects variable (TDAY) were run on PCREV, FS and all performance measures to test for effects of putative gaze control strategy on gaze behaviour and DS performance.

Greenhouse-Geisser corrections were employed in case of sphericity violations. Significant main effects were followed employing Bonferroni-Holm post hoc tests.

Target order was investigated as part of the participants’ chosen behavioural strategy. A chi-square test was used to determine whether the distribution of target order permutations selected by participants was different from what would be expected from chance. Subsequently, 1-sample proportion tests were used to determine which permutations were significantly different from chance at a *p* < 0.001.

K-means clustering analyses, which reveal the existence and separation of clusters in data, were employed using Matlab to separate the nave participants into two groups exhibiting either chasing or ambushing behaviours based on gaze control measures. K-means tests have been used to identify group membership in similar studies, one of which analysed the relationship between skill level and visual strategy in tennis^27^. Our measures were designed only to provide information about eye control during aiming to differentiate a ‘chasing’ vs. an ‘ambushing’ strategy. Therefore, in the present study, only a binary classification of participants was anticipated. This is in contrast to some applications of K-means clustering, where the number of clusters expected is unknown. Here, we simply use K-means as a way to label participants as employing either a chasing or ambushing strategy. The K-means tests in this study were thus all run with K = 2 and n = 22 on either a single variable (PCREV, or FS) or on two variables per training day (PCREV and FS together) to explore how participants fell into groups related to various measures of strategy.

## Results

### Performance improved in all participants

**Figure 2:**
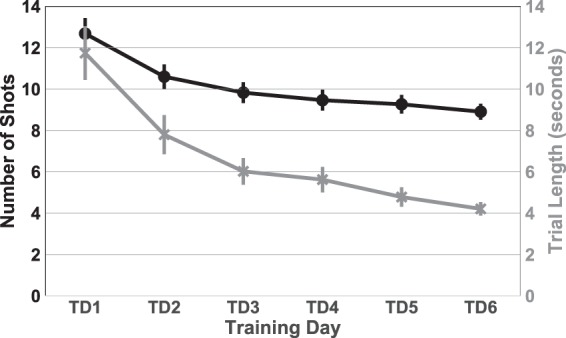
Group comparisons in two performance indices Average Number of Shots per Trial (black) and Average Time per Trial (grey). Error bars represent ±1 SEM. Notice that by the end of the training period, on average, our participants were completing a trial (five successful shots) in a little more than 4 seconds.

A RM ANOVA with training day (Training Days 1–6) as the within-subjects variable (TDAY) was run on the Trial Length data. The test revealed a significant result of TDAY, *F*(1.55, 32.64) = 55.34 *p* < 0.0001, *η*^2^ = 0.725 (see Fig. 2). Post hoc tests showed that days 1 and 2 were significantly different from all other days, followed by a stepwise decrease across the last four days.

A RM ANOVA with training day (Training Days 1–6) as the within-subjects variable (TDAY) was run on the Number of Shots data. The test revealed a significant result of TDAY, *F*(2.51, 52.86) = 60.84 *p* < 0.0001, *η*^2^ = 0.743 (see Fig. 2). Post hoc tests showed that days 1 and 2 were significantly different from all other days, followed by a stepwise decrease across the last four days.

A RM ANOVA with training day (Training Days 1–6) as the within-subjects variable (TDAY) was run on the Shot Accuracy data. The test revealed a significant result of TDAY, *F*(3.28, 68.78) = 5.41 *p* < 0.002, *η*^2^ = 0.2. Post hoc tests showed that days 1, 2 and 3 were significantly different from day 6, showing an increase in shot error distance across training. This indicates that our participants reduced Trial Length and number of shots per trial at the expense of accuracy.

### Personal strategy differed across participants

In order to characterise the participants’ strategies while performing the DS task, we looked at their gaze control strategies and choice of shot order.

#### Gaze control

We conducted separate K-means tests with K = 2 and n = 22 on each participant’s mean PCREV, mean FS and both measurements together for training days 3 to 6 in order to investigate how participants might fall into groups relative to gaze control metrics. For FS, we expected that smaller values would indicate chasing behavior and larger values would indicate ambushing behavior. For PCREV, we expected that larger values would indicate chasing behavior, and that smaller values would indicate ambushing behavior. The K-means tests we conducted on each metric individually did divide participants into groups along these lines, and the tests conducted on FS and PCREV simultaneously also indicated similar groupings. As the days progressed, the agreement between the groups revealed by the three tests increased. By Training Day 6, only one participant was classified differently by the K-means test on FS. Similarly, the classification of participants by PCREV only and PCREV and FS together were wholly consistent on Training Day 6. To investigate the behavior of participants across days, a K-means test on PCREV data pooled across Training Days 3–6 was also conducted. This test produced the exact same groups as the K-means test on FS and PCREV together on Training Day 6. A similar Pooled analysis of FS alone for Training Days 3–6 produced almost identical groups. Table 1 shows a summary of the participant groupings obtained from each test.

**Table 1:** Summary of K-means test groups for each test performed.

Test Description (K = 2, n = 22)	Ambushers (Participant Number)	Chasers (Participant Number)
TD3 FS	5,7,8,9,11,13,15,18,21	1,2,3,4,6,10,12,14,16,17,19,20,22
TD4 FS	5,8,9,11,13,15,18	1,2,3,4,6,7,10,12,14,16,17,19,20,21,22
TD5 FS	2,5,8,9,11,15,18,19,20	1,3,4,6,7,10,12,13,14,16,17,21,22
TD6 FS	2,5,8,9,11,15,18,20	1,3,4,6,7,10,12,13,14,16,17,19,21,22
TD3 PCREV	5,8,9,11,20	1,2,3,4,6,7,10,12,13,14,15,16,17,28,19,21,22
TD4 PCREV	8,9,13,15,18	1,2,3,4,5,6,7,10,11,12,14,16,17,19,20,21,22
TD5 PCREV	2,5,8,9,10,11,13,15,18,20	1,3,4,6,7,12,14,16,17,19,21,22
TD6 PCREV	2,5,8,9,10,11,13,15,18,20	1,3,4,6,7,12,14,16,17,19,21,22
TD3 [PCREV FS]	5,8,9,11,20	1,2,3,4,6,7,10,12,13,14,15,16,17,18,19,21,22
TD4 [PCREV FS]	5,8,9,11,13,15,18	1,2,3,4,6,7,10,12,14,16,17,19,20,21,22
TD5 [PCREV FS]	2,5,8,9,11,13,15,18,20	1,3,4,6,7,10,1214,16,17,19,21,22
TD6 [PCREV FS]	2,5,8,9,10,11,13,15,18,20	1,3,4,6,7,12,14,16,17,19,21,22
Pooled PCREV (TD3-TD6)	2,5,8,9,10,11,13,15,18,20	1,3,4,6,7,12,14,16,17,19,21,22
Pooled FS (TD3-TD6)	2,5,8,9,11,13,15,18,19,20	1,3,4,6,7,10,12,14,16,17,21,22

**Figure 3:**
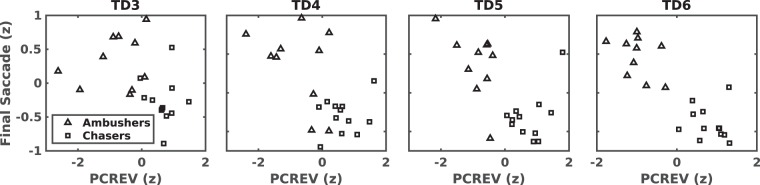
Mean PCREV and FS for each participant on Training Days 3–6. All measurements were normalised to z scores. Squares represent Chasers and triangles represent Ambushers as identified by a bivariate K-means test considering both FS and PCREV on day 6 with K = 2 and n = 22.

To visualize how the participants’ group membership evolved over the last four training days, Fig. 3 shows each participant’s mean PCREV and FS z-score as a function of day. Participant group membership in Fig. 3 was based on the the Training Day 6 K-means test on both FS and PCREV together.

Visual inspection of Fig. 3 supports the binary grouping of participants, with a clear gap between groups in both FS and PCREV emerging by the end of the training. It is also interesting to note that relative to the Training Day 6 [PCREV FS] classification, only one participant was in a different group on Training Day 5, and only 3 participants on Training Day 4. This suggests that a participant’s strategy was relatively stable once adopted. The consistency of groupings obtained from K-means tests on each metric alone with those obtained from the bivariate tests of PCREV and FS together suggest that it is possible to use either metric alone or both together to classify participants.

**Figure 4:**
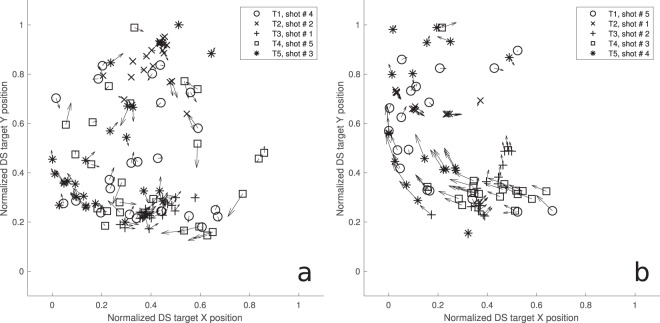
Dispersion of successful target shots for a typical chaser (**a**) and a typical ambusher (**b**) for days 5–6. Only trials executed in a particular target order are shown for clarity (chaser n = 24/40, ambusher n = 18/40). Arrow vectors represent the direction and relative speed of the target (longer line, higher speed) when it was shot. Note the broader dispersion, slower speeds and diverse directions for the chaser, suggesting that the participant tracked the targets across the full extent of the task space. Conversely, the ambusher waited for the targets to enter the left side of the task space.

**Figure 5:**
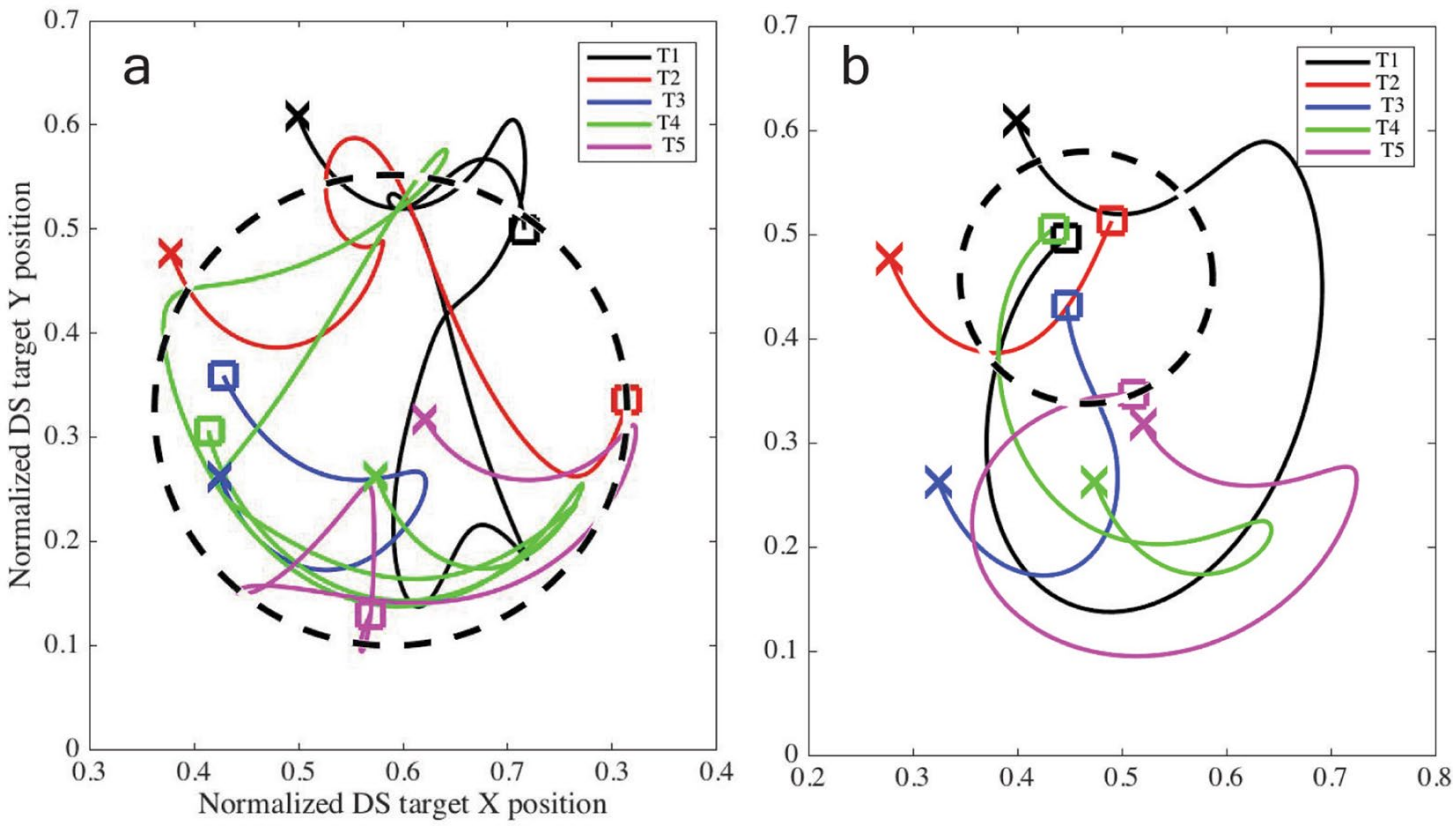
DS target trajectories in space for a typical chaser (**a**) and a typical ambusher (**b**) for training day 6, trial 20. Target locations at the beginning of the trial are denoted by an ‘x’ marker, while the square marker indicates the location where that target was eventually hit. Note the differences in the trajectory of each target. The ambusher’s choice of target order causes the DS target carrier to ‘spin’ allowing for most of the targets to be captured within a small area (dashed circle), while the chaser’s strategy involved capturing targets primarily at the ends of the DS target’s ‘arm’ pendular swing while preventing the DS from spinning. The dashed circles are centred at the mean in both dimensions of all successful shots for one individual trial, with the radius representing the largest deviation from the mean.

Therefore, based on the groups shown in Fig. 3, which represent the ‘TD6 [PCREV FS]’ results from Table 1, we labeled participants as either ambushers (n = 10) or chasers (n = 12). Figure 4 shows an example of the shot distribution in the task space for a typical participant of each group. Figure 5 shows the trajectory taken by each target during the final trial on the final day for a typical participant in each group, illustrating how targeting order and gaze strategy can produce vastly disparate possibilities for eventual target locations.

#### Shot order

**Figure 6:**
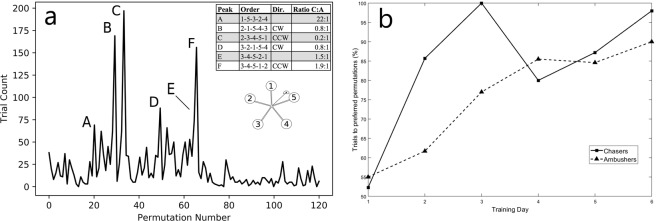
(**a**) Histogram of shot order for all participants. Inset shows the six most common shot permutations. Column Dir. shows the direction of shot order, CW = clockwise, CCW = counterclockwise. Column Ratio C:A represents the proportion of trials completed by each group, as confirmed by PCREV scores (below). DS target numbers displayed below the inset. (**b**) Selection per group of particular permutations (Group 1: A, E, F, Group 2: B, C, D) as a function of training day when only trials using permutations A-F are considered. The percentage of specific permutations favoured by each group increased as training progressed. C = Chasers, A = Ambushers.

There were 2,640 total successful trials (22 participants X 20 trials p/session X 6 sessions). A chi-square test was run on the observed frequency of selected shot order permutations in order to determine whether it differed from what would be expected from chance *χ*^2^(119) = 5,026.8, *p* < 0.01. Subsequent 1-sample proportion tests identified 15 permutations with high number of trials (*p* < 0.001, see Fig. 6(a)). Interestingly, four of the top six common permutations were executed either in a sequential clockwise or counterclockwise fashion. Since it is possible for participants to elicit a rotational movement in the DS by the order in which they shoot the targets, participants employing an ambushing strategy could, once they had learned the task, take advantage of this feature to pick off targets in sequence at nearly the same point in space.Therefore, we examined trials showing the two types of permutations listed in Fig. 6(a). We classified each of these as ‘chasing’ permutations in the cases A, E and F or ‘ambushing’ permutations in cases B, C and D. Across Training Days 3–6, participants employing ambushing permutations more often than chasing permutations aligned perfectly with the ambushing group identified by the K-means tests on gaze control described above.

In order to determine the development of shot order preference as a function of training day, we investigated trials in which participants engaged targets in the six most common shot orders. After downselecting the trials to only consider these six permutations, we calculated the percent of trials in which participants classified as chasers employed the ‘chasing’ permutations relative to ‘ambushing’ permutations. We employed a similar analysis for participants classified as ambushers to see how consistently they used sequential permutations. Figure 6(b) shows the proportion of trials taken by each group (25% of chaser trials and 33% of ambusher trials) to the permutations of their choice relative to those preferred by the other group. As can be seen, on Training Day 1, participants in both groups engaged in similar amounts of trials with ‘chasing’ and ‘ambushing’ permutations. However, as training progressed, each group exhibited clear preferences for particular shot orders.

#### Personal strategy showed correlates in gaze behaviour

**Figure 7:**
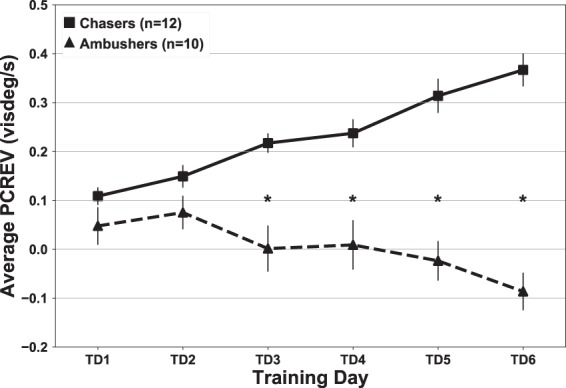
Average PCREV by group on training days 1–6. Error bars represent ±1 SEM. Significant differences between means are denoted with (*).

In order to confirm that the groups exhibited different gaze control strategies, we conducted a mixed-design ANOVA with Behavioural Strategy as the between-subjects variable (Chasers, Ambushers) and training day (Training Days 1–6) as the within-subjects variable (TDAY) was run on the PCREV data. The test revealed a significant difference of Behavioural Strategy *F*(1, 20) = 59.84 *p* < 0.0001, *η*^2^ = 0.75 as well as a significant interaction of Behavioural Strategy *TDAY, *F*(5,100) = 19.08 *p* < 0.0001, *η*^2^ = 0.49. Post hoc tests showed that the groups differed significantly from day 3 forward (Fig. 7), with chasers exhibiting higher values as training progressed. This indicates that chasers ‘caught up with’ targets more aggressively as training progressed by using higher eye velocities in the direction of target motion.

**Figure 8:**
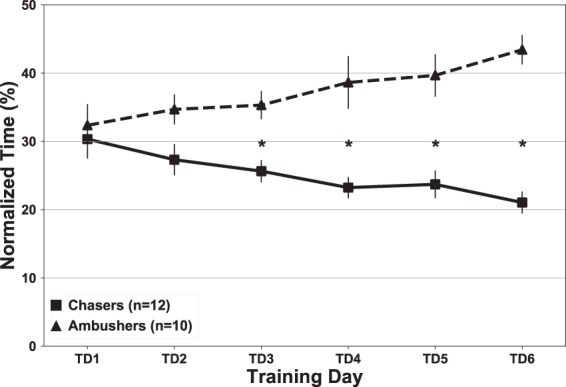
Comparison between chasers and ambushers on final saccade. Time was normalised over each pursuit attempt. Error bars represent ±1 SEM. Significant differences between means are denoted with (*).

Similarly, a mixed-design ANOVA with Behavioural Strategy as the between-subjects variable (Chasers, Ambushers) and training day (Training Days 1–6) as the within-subjects variable (TDAY) was run on the FS data in order to determine whether participants differed in the timing of their last saccade. The test revealed a significant difference of Behavioural Strategy *F*(1, 20) = 29.99, *p* < 0.0001, *η*^2^ = 0.6 as well as a significant interaction of Behavioural Strategy * TDAY, F(5,100) = 6.45 p < 0.0001, *η*^2^ = 0.24. Post hoc tests showed that the groups differed significantly from day 3 on (Fig. 8), with the chasers producing earlier FS as training progressed, suggesting that their gaze ‘locked’ onto a target’s path early in a pursuit attempt, and then followed it using smooth pursuit eye movements until the shot was taken. Ambushers, by contrast, showed later FS, which indicates the use of predictive eye movements to estimate the trajectory of the target. Their strategy aims to finally rest their gaze on the predicted location for their chosen target, and then waiting for the target to enter their visual field (Fig. 7).

Subsequent mixed-design ANOVAs with Behavioural Strategy as the between-subjects variable (Chasers, Ambushers) and training day (Training Days 1–6) as the within-subjects variable (TDAY) were conducted on all performance measures. There were no significant effects nor interactions. This indicates that gaze strategy did not have any effects on successful performance of the DS task.

## Discussion

The present study sought to characterise the behavioural strategies displayed by participants when performing the Death Star marksmanship task, which requires participants to track and capture targets moving in complex paths. Our results indicate that as participants learned to solve the DS task (as demonstrated by performance measures such as Trial Length and number of shots), they spontaneously adopted either a chasing or an ambushing strategy.

The DS simulation^18^ provides a realistic representation of the original target shooting task, with targets that move relatively unpredictably in two-dimensional space. In the present study, individuals improved at this task over a succession of days, including reductions in trial duration and number of shots (Fig. 2) across both ‘Chaser’ and ‘Ambusher’ groups, with no significant differences in performance between groups. Importantly, as Trial Length and number of shots decreased, missed shots were increasingly farther away from the intended target. This trade-off occurred in all participants.

This speed-accuracy trade-off is suggestive of Fitts’ Law^28^, which predicts that the movement time needed to capture a target scales with the log of the target’s ‘index of difficulty’, which depends on the target’s width and the distance the subject must travel to reach it. In fact, recent work has suggested that Fitts’ Law holds when describing the speed-accuracy trade-off in marksmanship during transitions between stationary targets^29^. Some studies outside the scope of marksmanship, like the work of Hoffman^30^ have attempted to modify Fitts’ Law to describe capture of moving targets, relying on the assumption that the target capture behaviour is dominated by a feedback loop closed around target position. This is in line with the ‘chasing behaviour’ we observed in one subset of our participants. Other studies, like the work of Tresilian^7^, have attempted to explain the speed-accuracy trade-off in moving target capture using a model that is independent of Fitts’ Law and assumes that the capture is performed primarily using feedforward or open-loop control, which roughly aligns with our observations of participants showing ‘ambush’ behaviour. However, Mottet and colleagues^31^ tested the case in which both a target and a pointer move concurrently towards each other when controlled by one or even two different participants. Their results showed that Fitts’ law held in all cases, leading the authors to conclude that the relevant variable is the total distance between the target and the pointer. In terms of the DS, this would suggest that a trade-off is to be expected whether the participant chases or ambushes a target, as we observed in our study.

While both chasing and ambushing strategies have been reported in studies of hand-eye coordination tasks involving target tracking and capture^7–12^, closer experimentation of the choice of participant behaviour into these two strategic categories is scarce. In a study of hand-eye coordination for target capture in the work of Mrotek and Soechting^14^, which involved participants moving a finger on a touch screen to capture a target moving in a periodic, but relatively unpredictable curvilinear path similar to the motion of one of the DS task’s targets, the authors did report that some participants seemed to have different strategies for target capture. Mrotek and Soechting^14^ reported that two of their subjects tended to ‘intercept’ the targets, while another tended to catch the target ‘from behind.’ This seems to suggest the presence of both chasing and ambushing behaviour as described in the present study, but the bifurcation and strength of participant engagement in chasing and ambushing was not a focus. Similarly, Land^32^ alludes to these “two forms of visuomotor control”, and suggests that they are “likely to be common features of many skilled eye-hand tasks”. In our study, we found that not only were the two strategies almost equally occurring in our participant pool (12 chasers, 10 ambushers), but that the ‘strength’ of the behavioural separation between groups employing each strategy increased as participants learned the task. This separation was supported by measures of both PCREV (Fig. 7) and FS (Fig. 8). One potential catalyst for this may lie in participants’ ability to learn to use target order to dictate whether target motion would be more conducive to either chasing or ambushing behaviour.

We did not expect individuals to show any preferences for particular permutations of shooting the targets (Fig. 6). Upon analysis, it became apparent that participants realised that by employing specific shot orders it was possible to make the DS more predictable. This would allow for repetitive oculomotor patterns, which are a fundamental component of precise target tracking^25^. Timing, combined with the physics of the movement (i.e. accounting for gravity, momentum, etc.) may have impacted the preference for certain shot orders. Our data suggest that participants favoured target shooting strategies that employed either a clockwise or counterclockwise sequence probably due to the fact that these sequences minimise the distance (in both physical and perceptual terms) between shots taken at successive targets. K-means clustering conducted on PCREV and FS measures returned two separate groups that corresponded perfectly with the division in participants who preferred popular sequential target orders over popular non-sequential orders. Based on their behavioural characteristics, we classified the participants as either ‘chasers’ or ‘ambushers’.

In terms of Shot Order, ambushers favoured sequential permutations that used the pendulum’s swing to cause the targets to enter a specific location in space where they could be picked off in rapid succession. Chasers, on the other hand, were more flexible about choice of strategy. For example, target sequence 1-5-3-2-4, employed mostly by chasers, typically resulted in reduced DS rotation by the counterbalancing of target weights, if executed with the correct timing of shots (Fig. 5).

Given that group membership in gaze control strategy was identified during data analysis, we did not have any a priori hypotheses regarding the source of such preferences. Interestingly, based on our participant survey regarding prior eye-hand coordination experience, it was noted that the subgroup of participants who had some expertise in visuomotor control (athletes or avid sportsmen) seemed to adopt the chasing strategy more commonly, although this trend did not approach significance according to a chi squared test. While this evidence is correlational, and thus cannot be interpreted as an assertion that learning to chase targets rather than ambush them is somehow ‘better’, it is possible that having prior eye-hand coordination experience enhances the ability to process the motion of a target of interest in relationship to other things, favouring a more flexible shooting strategy. Future studies will be needed to test this hypothesis.

An interesting interpretation of our results involves the recent theoretical views in eye-hand coordination of the phenomenon of Quiet Eye (QE). QE is a form of gaze control observed just before a critical action occurs in tasks that require aiming. Specifically, it refers to the protracted final fixation of the gaze to a single location or object in the visuomotor work-space. To be considered QE, this final fixation must remain within 3° of visual angle of the object, and persist for a minimum of 100 *ms*. QE has been used as a predictor of performance in tasks that require precise aiming^12,17,23^. QE has been reported in static shooting tasks like rifle shooting^16^ and in shotgun shooting of moving targets following a relatively consistent curvilinear path (e.g. clay birds)^11^. One possible interpretation of the difference in FS between the groups in our study (see Fig. 8) is that participants who exhibited earlier FS values may have had an increased QE duration in the moments leading up to a successful shot. If this were true, we would have expected the group with lower FS values (chasers) to show increased performance as training progressed and their FS values diverged from those of the ambusher group. However, the lack of a significant difference in overall task performance between groups suggests that interpreting FS as a proxy for QE would be insufficient to capture the differences in strategy our participants exhibited.

## Conclusions

The present study introduced individuals to a simulated competitive marksmanship task that involved shooting targets moving in relatively unpredictable trajectories. Our goal was to investigate a partially explored aspect of human motor control, namely, the choice of strategy to efficiently capture dynamic targets with complex motion. We found that two target-tracking strategies previously described in the literature appeared spontaneously (and developed as training progressed) at almost equal rates in this task. One strategy, which we call chasing, refers to an active pursuit tracking of a target throughout its motion until the participant decides to take a shot. The other, which we call ambushing, requires a participant to saccade to a pre-calculated location and wait for the target to reach it. Both strategies were equally effective at achieving high performance levels. Subsequent research may investigate the factors that influence an individual to have more chasing or ambushing behaviours.

## Data Availability

The datasets generated during and/or analysed during the current study are available at Open Science Framework: https://osf.io/gwrbz/?view_only=9e8667f3ab644a37aded5cc11444d9bf.

## References

[1] Krauzlis RJ (2005). The control of voluntary eye movements: new perspectives. The Neurosci..

[2] Lisberger SG (2010). Visual guidance of smooth-pursuit eye movements: sensation, action, and what happens in between. Neuron.

[3] Gawthrop P, Lakie M, Loram I (2008). Predictive feedback control and fitts–law. Biol. cybernetics.

[4] Kowler E, Martins AJ, Pavel M (1984). The effect of expectations on slow oculomotor control—iv. anticipatory smooth eye movements depend on prior target motions. Vis. research.

[5] Allen JS, Matsunaga K, Hacisalihzade S, Stark L (1990). Smooth pursuit eye movements of normal and schizophrenic subjects tracking an unpredictable target. Biol. Psychiatry.

[6] Leung H-C, Kettner RE (1997). Predictive smooth pursuit of complex two-dimensional trajectories demonstrated by perturbation responses in monkeys. Vis. research.

[7] Tresilian JR (2005). Hitting a moving target: perception and action in the timing of rapid interceptions. Percept. & Psychophys..

[8] Marinovic W, Plooy AM, Tresilian JR (2009). The utilisation of visual information in the control of rapid interceptive actions. Exp. Psychol..

[9] Land MF, McLeod P (2000). From eye movements to actions: how batsmen hit the ball. Nat. neuroscience.

[10] Diaz, G., Cooper, J. & Hayhoe, M. Memory and prediction in natural gaze control. *Philos. Transactions Royal Soc. B: Biol. Sci*. **368**, 20130064 (2013). 10.1098/rstb.2013.0064 PMC3758207 24018726

[11] Causer J, Bennett SJ, Holmes PS, Janelle CM, Williams AM (2010). Quiet eye duration and gun motion in elite shotgun shooting. Medicine & Sci. Sports & Exerc..

[12] Vickers JN, Adolphe RM (1997). Gaze behaviour during a ball tracking and aiming skill. Int. J. Sports Vis..

[13] Diaz G, Cooper J, Rothkopf C, Hayhoe M (2013). Saccades to future ball location reveal memory-based prediction in a virtual-reality interception task. J. vision.

[14] Mrotek LA, Soechting JF (2007). Target interception: hand–eye coordination and strategies. J. Neurosci..

[15] Danion FR, Flanagan JR (2018). Different gaze strategies during eye versus hand tracking of a moving target. Sci. reports.

[16] Janelle CM (2000). Expertise differences in cortical activation and gaze behavior during rifle shooting. J. Sport Exerc. psychology.

[17] Vickers JN, Lewinski W (2012). Performing under pressure: Gaze control, decision making and shooting performance of elite and rookie police officers. Hum. movement science.

[18] Brown AA (2017). Modeling and simulating the dynamics of the “death star” shotgun target. Sports Eng..

[19] López-Moliner J, Brenner E, Louw S, Smeets JB (2010). Catching a gently thrown ball. Exp. Brain Res..

[20] Garrido-Jurado S, Muñoz-Salinas R, Madrid-Cuevas FJ, Marín-Jiménez MJ (2014). Automatic generation and detection of highly reliable fiducial markers under occlusion. Pattern Recognit..

[21] Garrido-Jurado S, Munoz-Salinas R, Madrid-Cuevas FJ, Medina-Carnicer R (2016). Generation of fiducial marker dictionaries using mixed integer linear programming. Pattern Recognit..

[22] Kassner, M., Patera, W. & Bulling, A. Pupil: an open source platform for pervasive eye tracking and mobile gaze-based interaction. In *Proceedings of the 2014 ACM international joint conference on pervasive and ubiquitous computing: Adjunct publication*, 1151–1160 (ACM, 2014).

[23] Vine SJ, Moore LJ, Wilson MR (2014). Quiet eye training: The acquisition, refinement and resilient performance of targeting skills. Eur. J. Sport Sci..

[24] Sharpe JA, Zackon DH (1987). Senescent saccades: effects of aging on their accuracy, latency and velocity. Acta oto-laryngologica.

[25] Kowler E (2011). Eye movements: the past 25 years. Vis. research.

[26] Bahill AT, McDonald JD (1983). Smooth pursuit eye movements in response to predictable target motions. Vis. research.

[27] Murray NP, Hunfalvay M (2017). A comparison of visual search strategies of elite and non-elite tennis players through cluster analysis. J. sports sciences.

[28] Fitts PM (1954). The information capacity of the human motor system in controlling the amplitude of movement. J. experimental psychology.

[29] Brown, A. A. & Coelho, C. J. Modeling goal-directed movements in modern pistol competition. In *2017 IEEE International Conference on Systems, Man, and Cybernetics (SMC)*, 770–775 (IEEE, 2017).

[30] Hoffmann ER (1991). Capture of moving targets: a modification of fitts’ law. Ergonomics.

[31] Mottet D, Guiard Y, Ferrand T, Bootsma RJ (2001). Two-handed performance of a rhythmical fitts task by individuals and dyads. J. Exp. Psychol. Hum. Percept. Perform..

[32] Land MF (2009). Vision, eye movements, and natural behavior. Vis. neuroscience.

